# Supplementary material for: Fusing Mobile Phone Sensing and Brain Imaging to Assess Depression in College Students
Source: Front Neurosci. 2019 Mar 21;13:248. doi: 10.3389/fnins.2019.00248 (PMC6437560; doi:10.3389/fnins.2019.00248)
Supplement: Supplementary file 1 [file Data_Sheet_1.PDF]

Supplemental Material

(A)

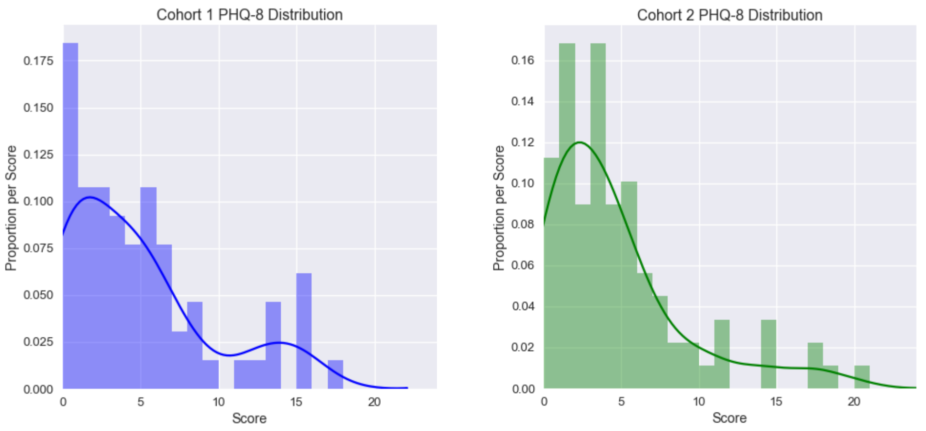

(B)

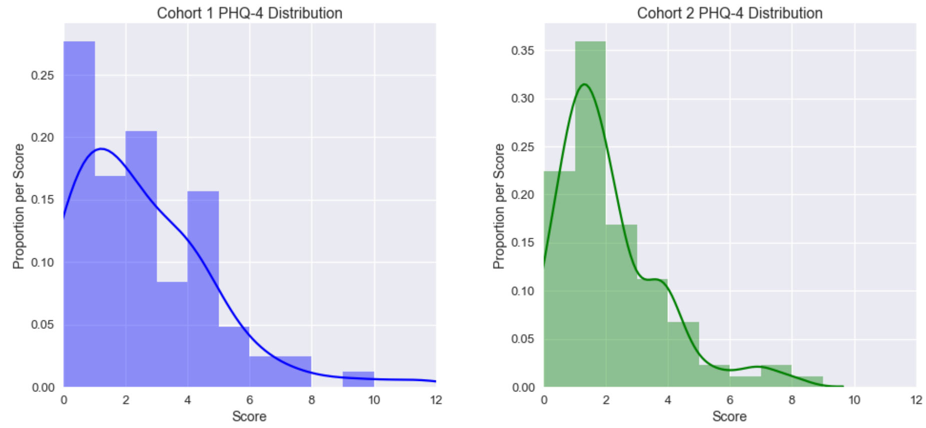

(C)

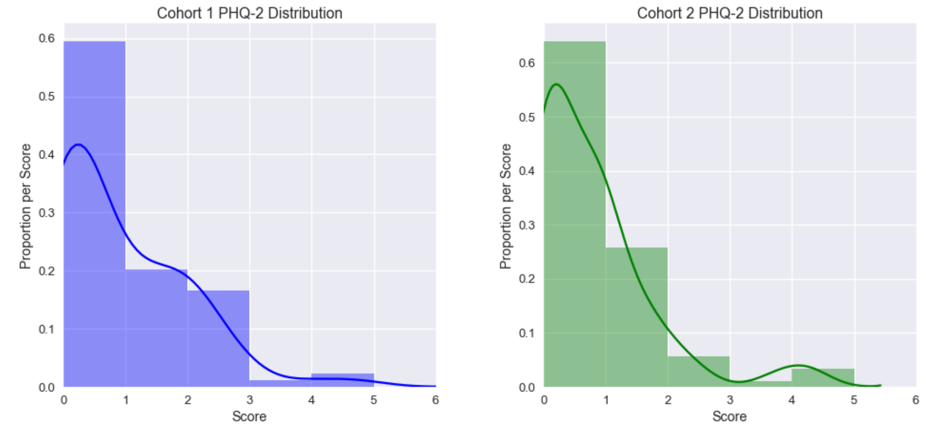

Figure S1. Distribution of depressive symptoms for (A) PHQ-8 taken before scan session, (B) PHQ-4 and (C) PHQ-2 taken through the StudentLife application during the academic term with Cohorts 1 on the left and 2 on the right.

(A)

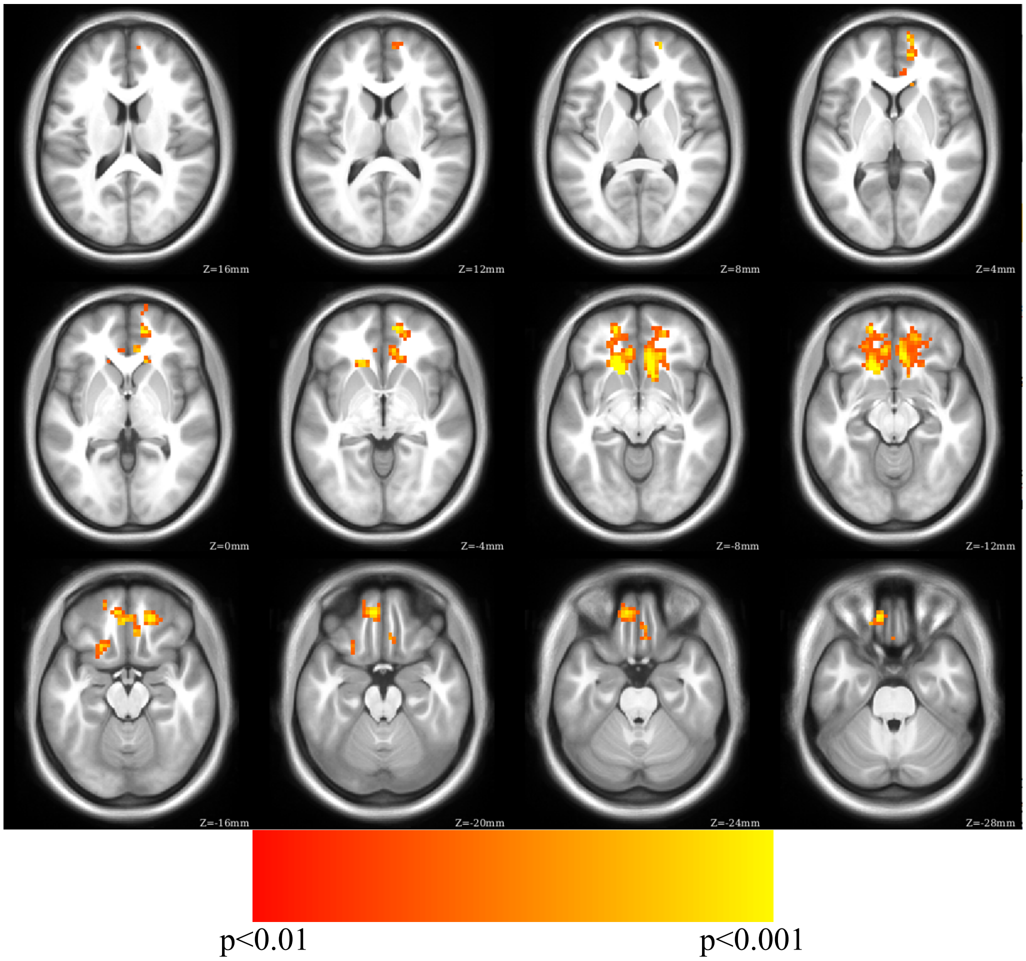

(B)

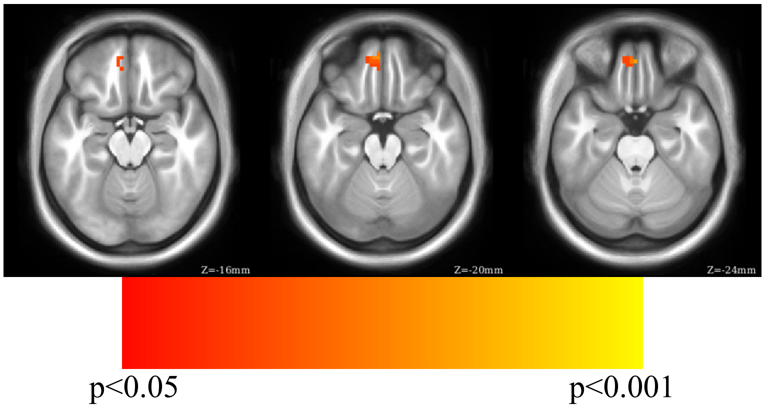

Figure S2. Exploratory analysis correlating sgCC RSFC seedmaps correlated with mean unlock duration in Cohort 1 (A) identified a cluster with a positive relationship to unlock duration in the ventromedial prefrontal cortex ($p<0.01$, volume corrected using ACF to 0.001, $k>449$) shown on axial slices. Replication of the relationship between unlock duration and sgCC connectivity between Cohorts 1 and 2 are observed in (B). The cluster identified in Cohort 1 showed a positive relationship with unlock duration was subsequently used to restrict the area interrogated in Cohort 2, showing positive clusters in ventral medial orbitofrontal cortex ($p<0.05$, volume corrected using ACF to $p<0.05$, $k > 38$).

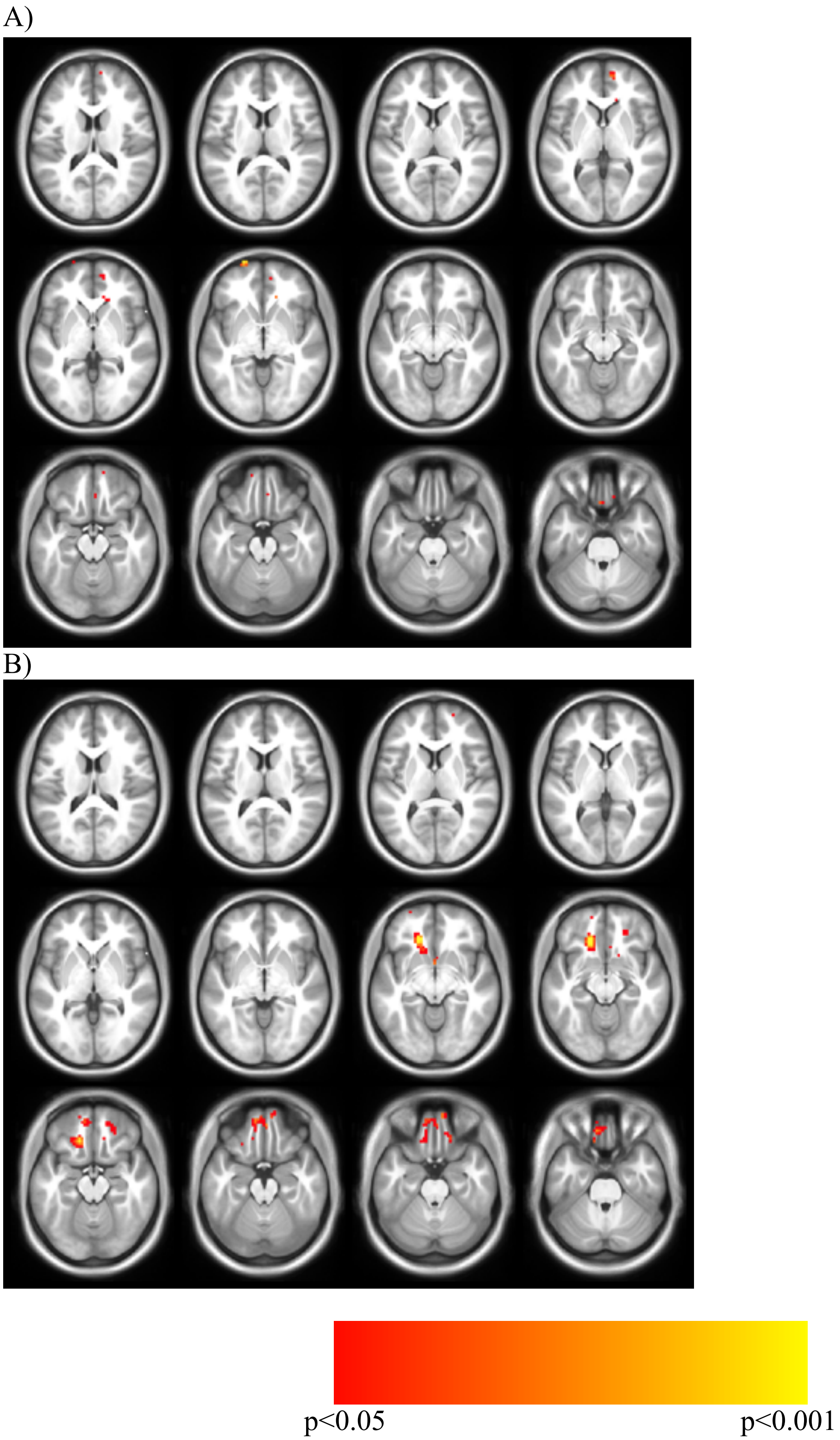

Figure S3. PHQ-4 regression for sgCC connectivity seedmaps for A) Cohort 1, B) Cohort 2 masked by the results from unlock duration in Cohort 1. No common regions were found between the two analyses.

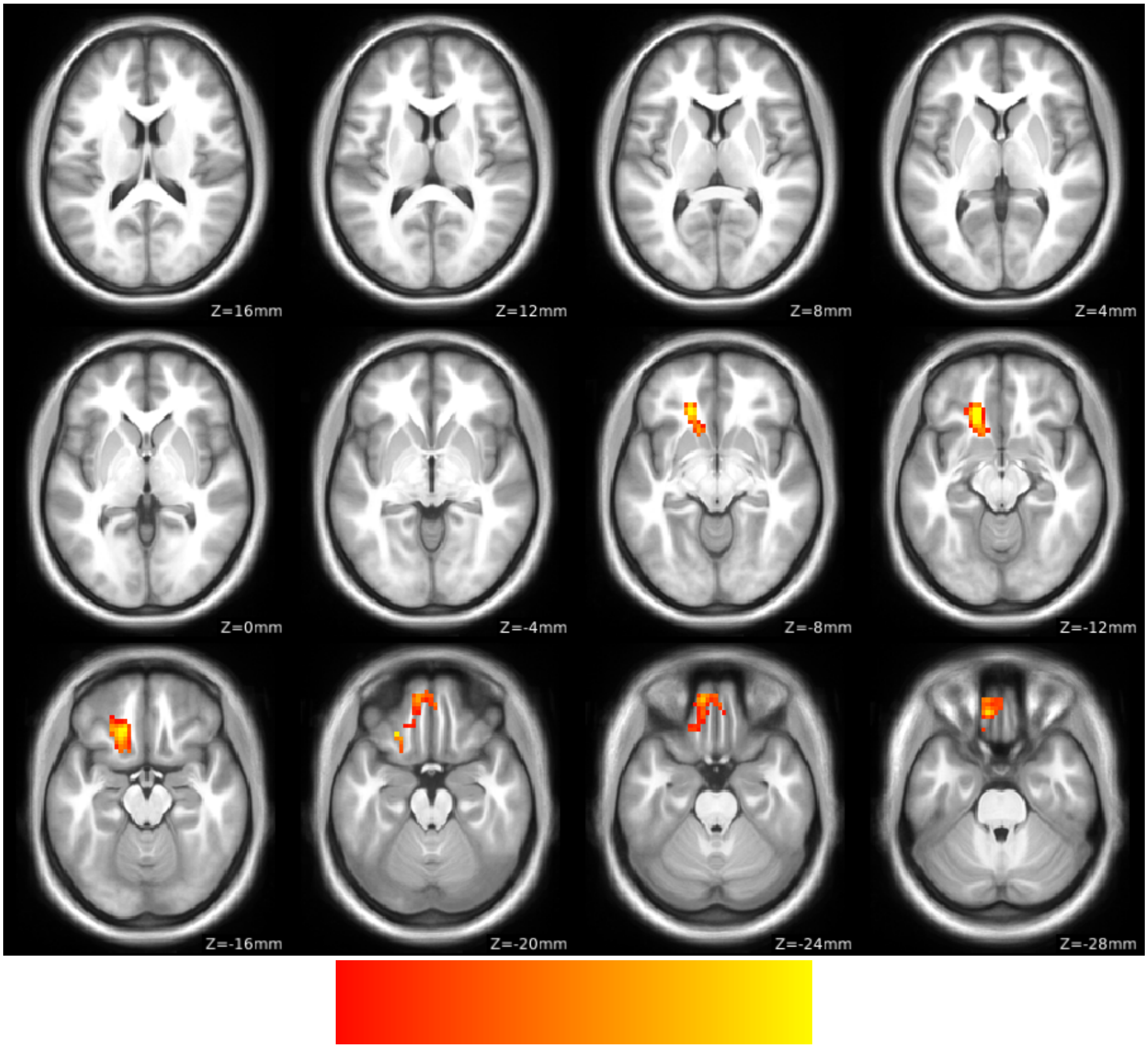

Figure S4. PHQ-2 regression for sgCC connectivity seedmaps for Cohort 2 masked by the results from unlock duration in Cohort 1, volume corrected to $p < 0.05$. No significant cluster were found in the Cohort 1 analysis and thus there was no overlap between the two analyses.

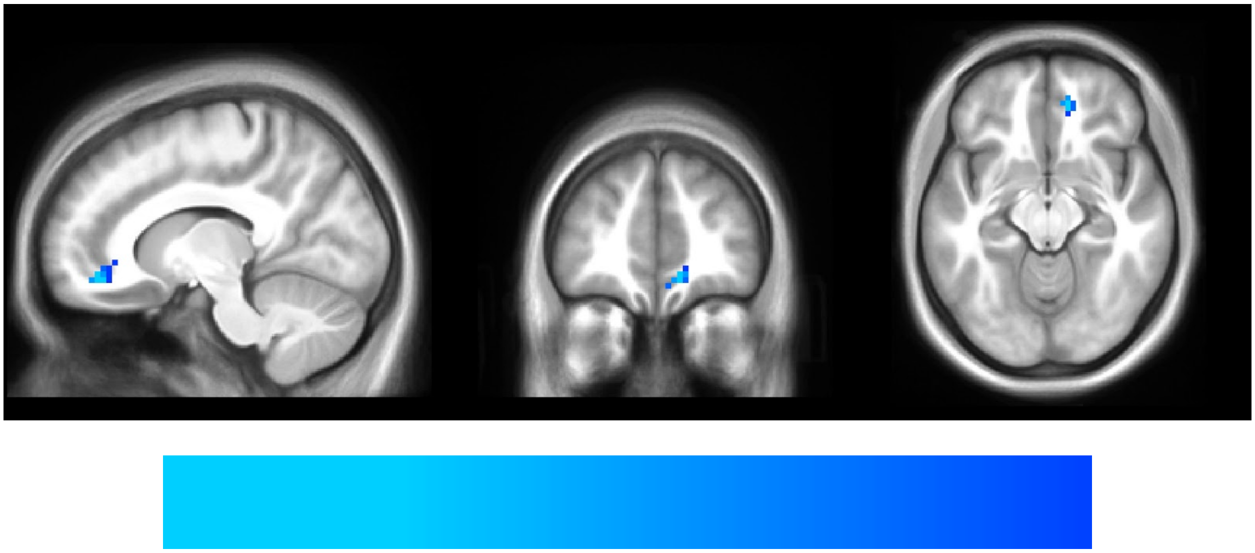

Figure S5. Subgenual cingulate cortex connectivity to medial orbitofrontal cortex is negatively correlated with the circadian similarity of phone movement in Cohort 2. The analysis was restricted to regions identified with the unlock duration analysis ($p<0.05$, volume corrected using ACF to $p<0.05$) . Sagittal, coronal and axial slices at MNI = 12, 45, -12. No significant regions within the unlock duration mask were identified in Cohort 1.

Table S1. Depression symptom severity separated by categories for both Cohorts 1 and 2.

PHQ-8 Score	Depression Severity	Cohort 1 N	Cohort 1 %	Cohort 2 N	Cohort 2 %
0 - 4	None-minimal	37	56.9	56	62.9
5 - 9	Mild	18	27.7	22	24.7
10 - 14	Moderate	5	7.7	7	7.9
15 - 19	Moderately severe	5	7.7	3	3.4
20+	Severe	0	0.0	1	1.1

Table S2. Exploratory analysis identifying peaks with a positive relationship from sgCC RSFC seedmaps correlated with phone-based surveys of depressive symptoms (EMA form of PHQ-4). Only clusters showing overlap with the Cohort 1 unlock duration analysis, with a positive relationship between PHQ-4 and connectivity with greater than 5 contiguous voxels are reported here. * signifies that cluster didn't pass volume correction.

**Cohort 1**					
**Best Estimate of Region**	**X**	**Y**	**Z**	**T**	**Extent**
Superior Frontal Gyrus*	-21	72	-3	2.99	7
Medial PFC*	9	57	3	2.31	7
**Cohort 2**					
**Best Estimate of Region**	**X**	**Y**	**Z**	**T**	**Extent**
Ventromedial PFC	-15	30	-12	3.71	139
	-9	51	-18	2.87	Subpeak
	3	48	-21	2.49	Subpeak
Anterior Medial OFC*	9	57	-24	2.38	7
Right OFC*	18	33	-24	2.11	5
Right OFC*	24	39	-15	1.87	13
	15	51	-15	1.72	Subpeak
**No Cohort Overlap Observed**					
